# Thermo-Exfoliated Graphite Containing CuO/Cu_2_(OH)_3_NO_3_:(Co^2+^/Fe^3+^) Composites: Preparation, Characterization and Catalytic Performance in CO Conversion

**DOI:** 10.3390/ma3010572

**Published:** 2010-01-20

**Authors:** Elena V. Ischenko, Ludmila Yu. Matzui, Snezhanna V. Gayday, Ludmila L. Vovchenko, Tatyana V. Kartashova, Vladyslav V. Lisnyak

**Affiliations:** Kyiv Taras Shevchenko National University, 64 Volodymyrska str., UA-01033, Kyiv, Ukraine; E-Mails: isch@voliacable.com (E.V.I.); matzui@univ.kiev.ua (L.Yu.M.); sgaydai@univ.kiev.ua (S.V.G.); vovch@univ.kiev.ua (L.L.V.); kartasheva@univ.kiev.ua (T.V.K.)

**Keywords:** Thermo-Exfoliated Graphite (TEG), catalytic CO conversion to CO_2_, CuO/Cu_2_(OH)_3_NO_3_, Powder X-ray Diffraction (PXRD), Scanning Electron Microscopy (SEM), Temperature Programmed Desorption Mass-Spectrometry (TPD MS)

## Abstract

Thermo-exfoliated graphite (TEG)/CuO/Cu_2_(OH)_3_NO_3_:(Co^2+^/Fe^3+^) composites were prepared using a wet impregnation method and subsequent thermal treatment. The physicochemical characterization of the composites was carried out by powder X-ray diffraction (PXRD), scanning electron microscopy (SEM) and Ar temperature-desorption techniques. The catalytic efficiency toward CO conversion to CO_2_ was examined under atmospheric pressure. Characterization of species adsorbed over the composites taken after the activity tests were performed by means of temperature programmed desorption mass-spectrometry (TPD MS). (TEG)/CuO/Cu_2_(OH)_3_NO_3_:(Co^2+^/Fe^3+^) composites show superior performance results if lower temperatures and extra treatment with H_2_SO_4_ or HNO_3_ are used at the preparation stages. The catalytic properties enhancements can be related to the Cu_2_(OH)_3_NO_3_ phase providing reaction centers for the CO conversion. It has been found that prevalence of low-temperature states of desorbed CO_2_ over high-temperature ones in the TPD MS spectra is characteristic of the most active composite catalysts.

## 1. Introduction

The conversion of carbon monoxide (CO) to carbon dioxide (CO_2_) at relatively low temperatures is an important process widely applied for environmental protection [1,2,3,4]. Nowadays, the search for CO conversion catalysts has been prompted by environmental needs to reduce emission levels of CO in the exhaust of fuel combustion processes [5,6,7,8,9]. Copper oxides are extensively utilized due to their good catalytic performance [10,11,12,13,14,15] and low fabrication costs. Among copper oxide catalysts, a composite consisting of CuO/Cu_2_(OH)_3_NO_3_ phases doped with Co^2+^ and Fe^3+^, further denoted as CuO/Cu_2_(OH)_3_NO_3_:(Co^2+^/Fe^3+^), recently obtained by us [16], exhibits sufficient activity in the CO conversion at relatively low temperatures giving a 100% conversion of CO to CO_2_ (*t*^100%^) at 373 K.

However, in order to decrease the amount of the catalyst used it seemed reasonable to prepare composite catalysts applying a proper carbon support to maintain the active phase in a highly dispersed state.

Different exfoliated graphite materials are characterized by high thermal resistance, flexibility, chemical stability, lightweight and compacting ability, the latter allows producing bulk composite materials of the prescribed shape [17,18]. Thermo-exfoliated graphite (TEG) obtained from the natural graphite by thermal shock [19] was chosen as a one of the promising component for the composite catalysts preparation. It should be noticed that notwithstanding a wide application of exfoliated graphites as gaskets, thermal insulators, fire-resistant composites [17,18] and sorbents for removing oil spills from water [20,21,22,23], scanty works report the exfoliated graphites as the base for composite catalysts or catalyst supports [24,25,26,27,28].

In the present study we report the preparation, physicochemical and structural characteristics of different TEG/CuO/Cu_2_(OH)_3_NO_3_:(Co^2+^/Fe^3+^) composites. Their characteristics were compared to find out the factors affecting the CO conversion efficiency.

## 2. Results and Discussion

### 2.1. Composite Preparation

Different procedures were used to prepare active CuO/Cu_2_(OH)_3_NO_3_:(Co^2+^/Fe^3+^)/TEG composites. Each procedure includes stages of impregnation and of drying of impregnated samples in the temperature interval from 353 to 573 K. The thermal treatment of impregnated TEG was performed to decompose metals nitrates to CuO oxide and Cu_2_(OH)_3_NO_3_ phase, both doped with Co^2+^ and/or Fe^3+^. The presence of Co^2+^/Fe^3+^ in the initial nitrate mixture is a necessary condition for crystallization of metastable monoclinic Cu_2_(OH)_3_NO_3_ phase from the solution.

### 2.2. Powder X-Ray Diffraction

**Figure 1:**
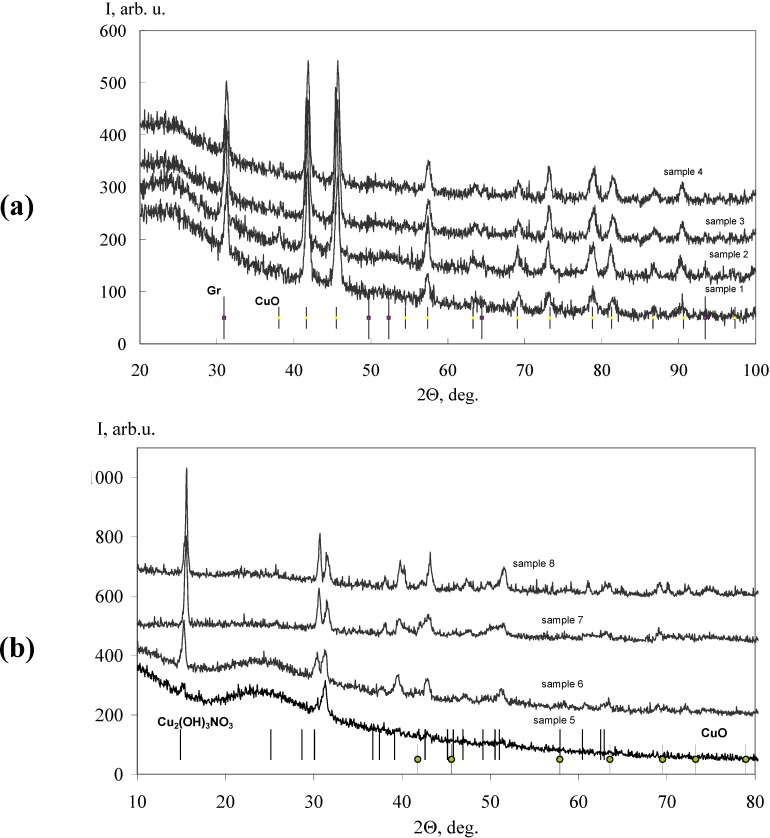
PXRD patterns of samples **1**–**4** (a) and **5**–**8** (b).

**Figure 2:**
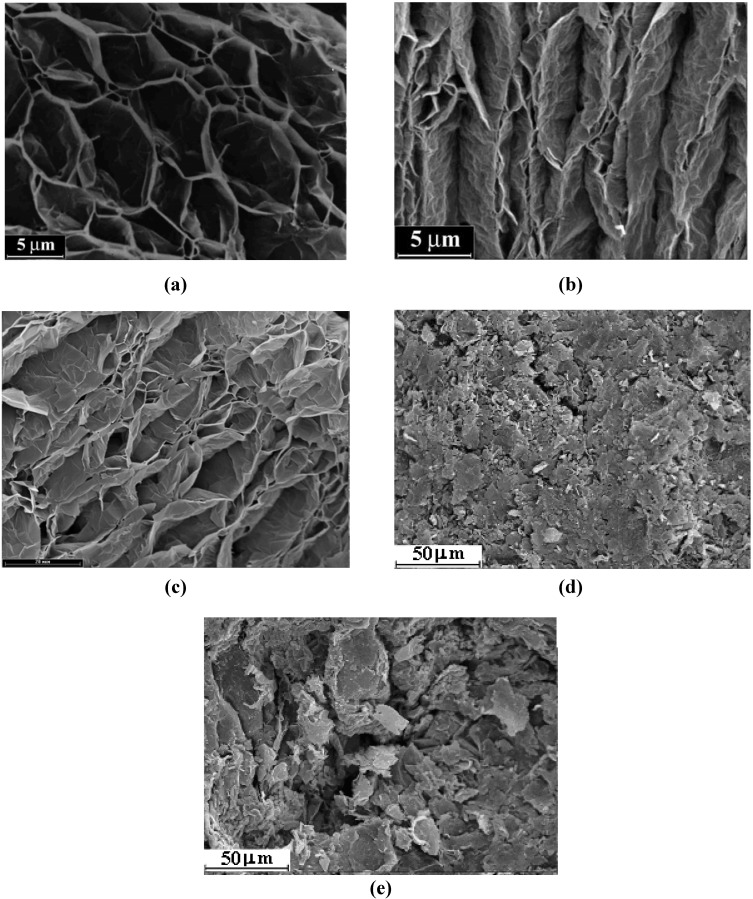
SEM micrographs of surface for (а) TEG; (b) TEG-H_2_SO_4_, (c) TEG-HNO_3_; (d, e) TEG/CuO/Cu_2_(OH)_3_NO_3_:(Co^2+^/Fe^3+^) composites **7**, **8**, respectively.

PXRD patterns of the composites (Figure 1 and Figure 2) show crystalline monoclinic CuO phase peaks (*a* = 0.4690(3) nm, *b* = 0.5133(8) nm, *c* = 0.3431(6) nm, *β* = 99.76(7)°) and monoclinic metastable Cu_2_(OH)_3_NO_3_ phase ones (*а* = 0.5613(2) nm, *b* = 0.6956(1) nm, *c* = 0.6098(6) nm, *β* = 92.35(1)°); both phases are doped with Co^2+^ and/or Fe^3+^. The diffraction peaks are in good accordance with JCPDS-PDF-2 card No 65–2309 (CuO: *a* = 0.4687 nm, *b* = 0.5114 nm, *c* = 0.3417 nm, *β* = 99.49°) and with JCPDS-PDF-2 card No 75–1779 (Cu_2_(OH)_3_NO_3_: *a* = 0.5605 nm, *b* = 0.6929 nm, *c* = 0.6087 nm, *β* = 94.48°), respectively. It can be clearly seen from the Figure 1 that the samples **1**–**4** dried at temperatures above 473 K contain a single crystalline phase CuO:(Co^2+^/Fe^3+^), in contrast to the samples **5**–**8**, dried at temperatures below 473 K (Figure 2). The PXRD patterns of **5**–**8** show formation of biphase Cu_2_(OH)_3_NO_3_:(Co^2+^/Fe^3+^) and CuO:(Co^2+^/Fe^3+^) mixtures. The PXRD patterns of **5**–**8** contain no graphite phase in contrast to that for **1**–**4**, exhibiting residual *h*-graphite (according to JCPDS-PDF-2 card No 41–1487), which main (002) peak was positioned at 2*θ* = 31.05°–31.2° (Figure 1). The PXRD patterns of **5**–**8** confirm that the complete graphite exfoliation has occurred (Figure 2).

### 2.3. Catalytic Test, Specific Surface Area and Scanning Electron Microscopic Characterization

Prior to analysis of the catalytic test results obtained for the composites, it should be noted that the pure TEG itself shows a certain catalytic activity in the conversion of CO to CO_2_. A CO_2_ increase in the inlet gas mixture was registered at relatively low temperatures from 543 to 673 K and 90% conversion of CO to CO_2_ is reached at 673 K. The composites show higher activity then the pure TEG (*t*^90%^ = 673 K), their temperatures at 100% conversion of CO to CO_2_ (*t*^100%^) are below 673 K (Table 1).

It was found that the temperature of the thermal treatment used to decompose the metals nitrate sediments supported over the TEG influences the catalytic activity of composites noticeably. The highest activity was obtained for the composites treated at a temperature below 473 K; treatment of the composites at higher temperatures suppresses the activity. Consequently, one can suppose that the presence of Cu_2_(OH)_3_NO_3_:(Co^2+^/Fe^3+^) phase (thermostable up to 473 K) shows a strong influence on the CO conversion activity. Wet impregnation under ultrasonic agitation increases the surface area and the activity of the composites [Table 1, samples (**5**) and (**6**)]. The activity of the composites increases with the amount of CuO/Cu_2_(OH)_3_NO_3_:(Co^2+^/Fe^3+^), reaching a maximum at 57 mass % (Table 1).

**Table 1:** Surface area (*S*_sp_), the amount of CuO/Cu_2_(OH)_3_NO_3_:(Co^2+^/Fe^3+^) (*A*), phases determined from PXRD data (*B*), the temperature at 100% conversion of CO to CO_2_ (*t*^100%^), the peak temperature (*T*_d_) and energy of the CO_2_ (*m*/*z* 44) desorption (*E*_d_) from the TPD MS data.

Sample No	*S*_sp_ (m^2^·g^–1^)	*A* (mass %)	*B*	*t*^100%^ (K)	*T*_d_ (K)			*E*_d_ (kJ·mol^–1^)		
					desorbed CO_2_ states					
					*α*_1_	*α*_2_	*α*_3_	*α*_1_	*α*_2_	*α*_3_
1	4	47	CuO/Graphite	528	–	514	540, 588, 678	–	146	153, 167, 193
2	10	51	CuO/Graphite	483	–	520	588, 683	–	147	167, 195
3	4	48	CuO/Graphite	488	–	–	539, 583, 640	–	–	153, 166, 183
4	10	52	CuO/Graphite	468	373	475	543	105	134	154
5	10	15	CuO/Cu_2_(OH)_3_NO_3_	513	–	440, 476, 513	553, 638, 678	–	124, 134, 145	157, 182, 194
6	16	56	CuO/Cu_2_(OH)_3_NO_3_	438	–	498	533, 588	–	141	151, 167
7	20	51	CuO/Cu_2_(OH)_3_NO_3_	456	–	503	593, 703	–	142	169, 201
8	25	57	CuO/Cu_2_(OH)_3_NO_3_	403	–	513	603, 693	–	145	172, 198

According to the PXRD data the treatment mentioned in [19] can partially transform natural graphite into TEG. So, the main attention should be paid to the samples free of the graphite residues, which were prepared from the TEG, additionally treated with acids and subjected to the subsequent thermal shock at 1,073 K, further denoted as TEG-H_2_SO_4_ and TEG-HNO_3_.

For estimation of the effect of extra treatment with acids, the evolution of microtextural structure of the samples surfaces and specific surface area are analyzed and discussed below. SEM micrographs display the surface texture of TEG, TEG-H_2_SO_4_ and TEG-HNO_3_ (Figures 2a–c).

Comparing these micrographs, one can clearly see that the extra treatment with acids leads to significant changes in the microtextural structure and morphology of the TEG surface. According to the Ar temperature-desorption data, the thermo-exfoliation is accompanied by increase of *S*_sp_ from 2 m^2^·g^–1^ for the natural graphite to 19 m^2^·g^–1^ for the TEG obtained.

The extra impregnation and subsequent thermal treatment increase *S*_sp_ to 70 m^2^·g^–1^ and 26 m^2^·g^–1^, for TEG-H_2_SO_4_ and TEG-HNO_3_, correspondingly. The TEG treatment with acids develops a microtextural structure due to intensive decomposition of acids residues over TEG surface at thermal shock. It is observed that many pores with regular walls, found for the TEG surface, transform into pores which inner and outer-walls showed a concentric texture or even pore destruction occurs (Figure 2c). This observation comports with results of SEM examination of [29,30] illustrating morphological and microstructural changes at the exfoliation of TEG. From comparison of SEM micrographs of [29,30] and that presented one can suggest complete TEG exfoliation. The PXRD patterns of the TEG-H_2_SO_4_ and TEG-HNO_3_ exhibit no peaks from the basal plane of *h*-graphite, indicating complete exfoliation.

The extra treatment of TEG with H_2_SO_4_ just increases *S*_sp_ to 70 m^2^·g^–1^, but the followed operations used for the TEG/CuO/Cu_2_(OH)_3_NO_3_:(Co^2+^/Fe^3+^) composite preparation suppress *S*_sp_ to 20 m^2^·g^–1^. Figure 2c shows that CuO/Cu_2_(OH)_3_NO_3_:(Co/Fe) covers the surface of TEG-H_2_SO_4_, blocking the surface pores (see Figure 2d) and diminishing *S*_sp_ three-fold. In contrast to the samples described above, *S*_sp_ of TEG-HNO_3_ (found to be 26 m^2^·g^–1^) remains constant after wet impregnation with nitrate solution and subsequent thermal treatment. The TEG microtextural structure is clearly seen on the SEM micrograph (Figure 2e) due to higher dispersion of CuO/Cu_2_(OH)_3_NO_3_:(Co/Fe) over TEG impregnated with HNO_3_.

The composites prepared from the TEG-H_2_SO_4_ and TEG-HNO_3_ show higher activity than that obtained from the TEG containing graphite residues (Table 1). Among the composite samples examined the highest content of the TEG/CuO/Cu_2_(OH)_3_NO_3_:(Co^2+^/Fe^3+^) component is registered for **8**, possessing the highest catalytic activity (*t*^100%^ = 403 K). On the other hand, the relative content of Cu_2_(OH)_3_NO_3_ prevails over CuO phase in the sample **8** for the greatest extent. Comparing results of X-ray diffraction study and *t*^100%^ values, one can conclude that the catalytic activity of the composites might depend on Cu_2_(OH)_3_NO_3_ and/or CuO content, moreover among the composites studied the ones, richest with Cu_2_(OH)_3_NO_3_ phase, are characterized by the highest activity.

### 2.4. Characterization of Adsorbed Species by TPD MS after CO Conversion

**Figure 3:**
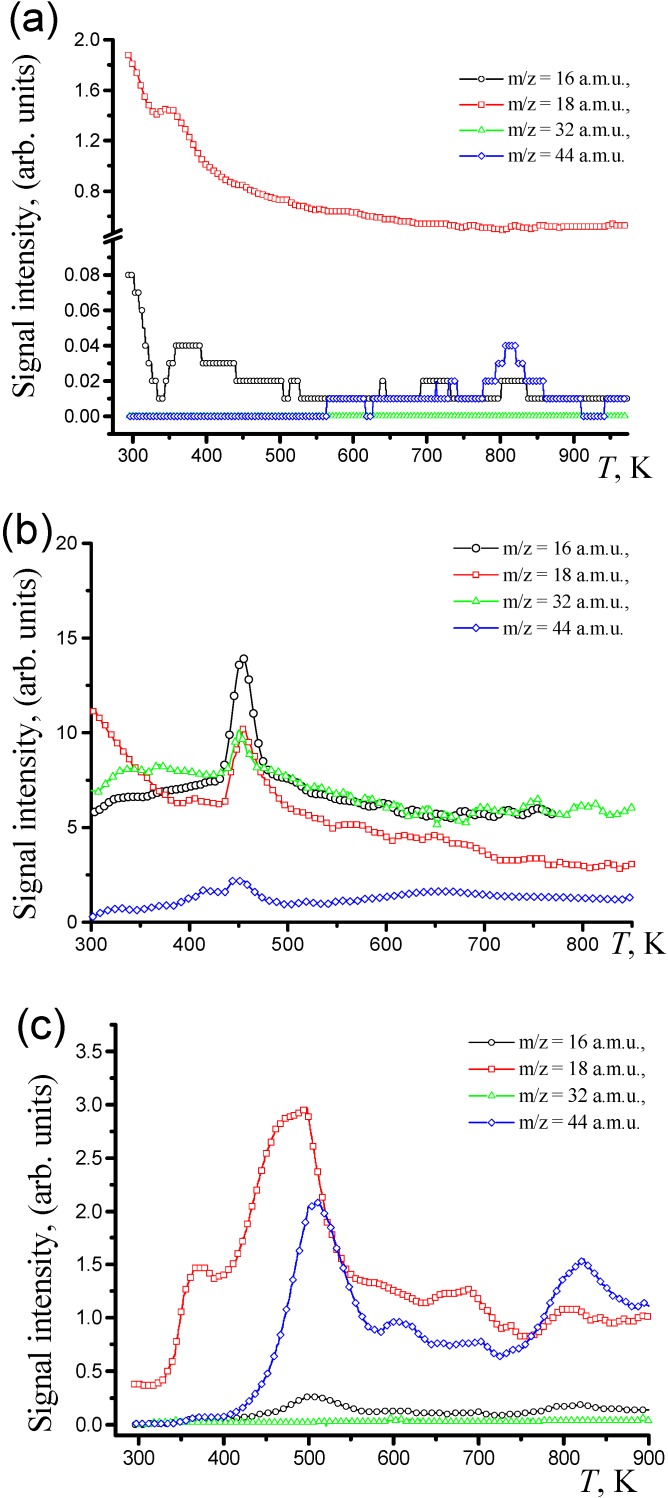
TPD MS profiles of different molecular ions taken after CO conversion to CO_2_ for: (a) TEG; (b) CuO/Cu_2_(OH)_3_NO_3_:(Co^2+^/Fe^3+^); (c) TEG/CuO/Cu_2_(OH)_3_NO_3_:(Co^2+^/Fe^3+^) referring to the sample **8**.

Prior to the TPD MS characterization of species adsorbed over the TEG/CuO/Cu_2_(OH)_3_NO_3_:(Co^2+^/Fe^3+^) composites, TPD MS profiles for the pure TEG were taken after the CO conversion recorded. The TPD MS profile of *m*/*z* = 18 a.m.u. (H_2_O^+^) was registered and no molecular oxygen profile was observed in the TPD MS spectrum of TEG (Figure 3a). In the TPD MS profile of *m*/*z* = 16 a.m.u. (O^+^) a peak of small intensity with was detected. The peak temperature of desorption (*T*_d_) equals 373 K. A typical TPD MS spectrum of the CuO/Cu_2_(OH)_3_NO_3_:(Co^2+^/Fe^3+^) composite taken after the CO conversion is presented in the Figure 3b.

The TPD MS profile of *m*/*z* = 18 a.m.u. (H_2_O^+^) shows an intensive symmetric peak corresponding to a dissociative desorption of water from the surface of the composite with *T*_d_ < 723 K. TPD MS profiles of CO_2_^+^ and O_2_^+^ are both of asymmetric character, meaning that the molecules desorb without dissociation. It was shown in previous studies [16] that the catalytic activity in the CO conversion for the bulk CuO/Cu_2_(OH)_3_NO_3_:(Co^2+^/Fe^3+^) composites correlates with intensity and area of peaks referring to atomic oxygen and CO_2_ in the TPD MS spectra.

Significant amounts of atomic oxygen and insignificant amounts of CO_2_ are found in all spectra of highly active catalysts. It is clearly seen from the comparison of the TPD MS spectra (Figure 3c and Figure 3b) that *T*_d_ for peaks with *m*/*z* = 16 (O^+^), 18 (H_2_O^+^), 32 (O_2_^+^) and 44 (CO_2_^+^) a.m.u. of the CuO/Cu_2_(OH)_3_NO_3_:(Co^2+^/Fe^3+^)/TEG composite is shifted to higher temperatures with respect to that registered for the bulk CuO/Cu_2_(OH)_3_NO_3_:(Co^2+^/Fe^3+^) sample. No profile of adsorbed molecular CO^+^ (*m*/*z* = 28) was detected for any of the samples taken after CO oxidation, so it is logical to conclude that CO adsorbed on surface oxygen atoms of the oxides is found in a relatively strong bonded state. Consequently, only the state of CO_2_ adsorbed species is discussed hereafter.

The peak temperature of the CO_2_ (*m*/*z* 44) desorption (*T*_d_) and respective desorption energy (*E*_d_) of the CO_2_ states for TEG/CuO/Cu_2_(OH)_3_NO_3_:(Co^2+^/Fe^3+^) composites are listed in Table 1. By the *T*_d_ and *E*_d_ the CO_2_ states can be assigned to physicosorbed CO_2_ state *α*_1_ with 102 ≤ *E*_d_(*α*_1_) ≤ 105 kJ·mol^–1^ at temperatures *T*_d_ ≤ 373 K (observed rarely as a peak of low intensity); to strongly chemisorbed states – *α*_2_ with 108 ≤ *E*_d_(*α*_2_) ≤ 148 kJ · mol^–1^ at 383 K ≤ *T*_d_ ≤ 523 K; *α*_3_ with 150 ≤ *E*_d_(*α*_1_) ≤ 222 kJ · mol^–1^ at 523 K ≤ *T*_d_ ≤ 773 K.

The peak referring to *α*_1_ state is observed for **4**, however this peak is absent in the TPD MS profile for the other samples. As could be seen from the Table 1, the low-temperature *α*_1_ state most probably can not act in the CO conversion, so an attention should be paid to interrelation of *α*_2_ and *α*_3_ states registered at the temperatures close to *t*^100%^. In our opinion, *α*_3_ can act in the reaction, but higher *E*_d_, as compared with *α*_2_ state, leads to the decrease of catalytic activity for the most of composites examined.

That is why the peaks referring to *α*_2_ and *α*_3_ should be compared, estimating ratios of their relative intensities [*I*(*α*_3_):*I*(*α*_2_) and areas (*S*(*α*_3_):*S*(*α*_2_)].

(1) If *I*(*α*_3_) >> *I*(*α*_2_) and *S*(*α*_3_) >> *S*(*α*_2_), the major amount of CO_2_ is bounded strongly with the surface for **1** and **5** and the later correlates with low CO conversion observed.

(2) In the case of close intensity and area of the desorption peaks both states participate in the stages of the CO conversion, this leads to moderate catalytic activity observed for **4** and **6**.

(3) If peak refers to *α*_2_ state is absent in the TPD MS profile, CO_2_ is strongly chemisorbed in the near-surface layer, suppressing the catalytic reaction. The later assumption is in accord with high *t*^100%^ values registered for **2** and **3**.

(4) On the other hand, *I*(*α*_2_) >> *I*(*α*_3_) is observed in the TPD MS profiles of **7** and **8**. The peak intensity is highest for **8**; the latter exhibits the highest catalytic activity. So, one can suggest that the main influence in the CO conversion to CO_2_ is contributed by *α*_2_ state.

## 3. Experimental Section

### 3.1. Reagents

#### 3.1.1. Thermo-Exfoliated Graphite

The TEG was prepared from natural graphite (Zavalievskoe coal-field, Ukraine) with lamellae of 100–200 mkm. This natural graphite was impregnated with 63 wt % H_2_SO_4_ and subjected to a thermal shock at 1,073 K within 12 sec. to obtain the TEG according to the methodology of [19].

#### 3.1.2. Nitrate Mixture

Aqueous solution of Cu^2+^/Co^2+^/Fe^3+^ nitrate mixture, used for the impregnation, was prepared by dissolving 1 g of mixture containing preset quantities of Cu, Co and Fe metals (all from Alfa-Aesar, reagent quality) in 15 mL of 12 M nitric acid (high purity grade, Aldrich). The metals quantities were calculated on Cu:Co:Fe ratio 90.25:4.75:5 (in mass %) in the resulted composite, obtained by evaporation of mentioned solution at 373 K up to formation of a wet sediment over the TEG. The Cu^2+^/Co^2+^/Fe^3+^ nitrate mixture concentration was calculated on a certain content of CuO/Cu_2_(OH)_3_NO_3_:(Co^2+^/Fe^3+^) in the resulted CuO/Cu_2_(OH)_3_NO_3_:(Co^2+^/Fe^3+^)/TEG composite.

### 3.2. Composite Preparation

*Sample 1.* The TEG was wet impregnated by the nitrates mixture for 6 h, dried for 4 h at *T* < 373 K in air and thermal shocked at 1,073 K for 12 sec. The Cu^2+^/Co^2+^/Fe^3+^ nitrate mixture concentration was calculated on 47 mass % content of CuO/Cu_2_(OH)_3_NO_3_:(Co^2+^/Fe^3+^) in the resulted composite.
*Sample 2.* The TEG was wet impregnated by the nitrates mixture for 6 h, dried for 4 h at *T* < 373 K in air and thermally treated at 473 K for 4 h. The Cu^2+^/Co^2+^/Fe^3+^ nitrate mixture concentration was calculated on 51 mass % content of CuO/Cu_2_(OH)_3_NO_3_:(Co^2+^/Fe^3+^) in the resulted composite.
*Sample 3.* The TEG was wet impregnated by the nitrates mixture with assistance of ultrasonic agitation using an ultrasonic disperser UZDN-2T operating at 10 kHz for 6 h. The impregnated TEG was dried for 36 h at *T* < 373 K in air and thermally shocked at 1,073 K for 12 sec. The Cu^2+^/Co^2+^/Fe^3+^ nitrate mixture concentration was calculated on 48 mass % content of CuO/Cu_2_(OH)_3_NO_3_:(Co^2+^/Fe^3+^) in the resulted composite.
*Sample 4.* The TEG was wet impregnated by the nitrates mixture with assistance of ultrasonic agitation (10 kHz) for 6 h. The impregnated TEG was for 4 h at *T* < 373 K in air and thermal treated at 473 K for 4 h. The Cu^2+^/Co^2+^/Fe^3+^ nitrate mixture concentration was calculated on 52 mass % content of CuO/Cu_2_(OH)_3_NO_3_:(Co^2+^/Fe^3+^) in the resulted composite.
*Sample 5.* The TEG was wet impregnated by the nitrates mixture for 4 h without assistance of ultrasonic agitation (10 kHz). The Cu^2+^/Co^2+^/Fe^3+^ nitrate mixture concentration was calculated on 15 mass % content of CuO/Cu_2_(OH)_3_NO_3_:(Co^2+^/Fe^3+^) in the resulted composite. The impregnated TEG was leached with distilled water, dried at 573 K for 36 h in air and thermally treated at 453 K for 1 h.
*Sample 6.* The TEG was wet impregnated by the nitrates mixture with assistance of ultrasonic agitation (10 kHz) for 4 h. The Cu^2+^/Co^2+^/Fe^3+^ nitrate mixture concentration was calculated on 56 mass % content of CuO/Cu_2_(OH)_3_NO_3_:(Co^2+^/Fe^3+^) in the resulted composite. The impregnated TEG was leached with distilled water, dried at 453 K in air and thermally treated at 453 K for 1 h.
*Sample 7.* The TEG was impregnated with 63 wt % H_2_SO_4_ for 12 h. The TEG impregnated with acid was leached with distilled water up to filtrate pH = 5.5; dried at temperatures from 373 to 393 K for 8 h, and, finally, thermally shocked at 1073 K for 12 sec. The resulting TEG-H_2_SO_4_ was wet impregnated with the nitrates mixture under ultrasonic agitation (10 kHz) for 4 h, dried at 373 K and thermally treated at 453 for 1 h. The Cu^2+^/Co^2+^/Fe^3+^ nitrate mixture concentration was calculated based on a 51 mass % content of CuO/Cu_2_(OH)_3_NO_3_:(Co^2+^/Fe^3+^) in the resulting composite.
*Sample 8.* The TEG was impregnated with 68 wt % HNO_3_ for 12 h. The TEG impregnated with acid was leached with distilled water up to filtrate pH = 5.5; dried at temperatures from 373 to 393 K for 8 h in air, and, finally, thermally shocked at 1,073 K for 12 sec. Resulted TEG-HNO_3_ were wet impregnated by the nitrates mixture under ultrasonic agitation (10 kHz) for 4 h, dried at 373 K and thermally treated at 453 for 1 h. The Cu^2+^/Co^2+^/Fe^3+^ nitrate mixture concentration was calculated on 57 mass % content of CuO/Cu_2_(OH)_3_NO_3_:(Co^2+^/Fe^3+^) in the resulted composite.

### 3.3. Composite Characterization

Powder X-ray diffraction (PXRD) patterns were collected in *θ–θ* mode using a DRON-4-07 diffractometer (filtered CoКα_1_-radiation, *λ* = 1.78897 Å, graphite monochromator). Samples were ground to powder before the analysis. The diffraction data was collected in a continuous scan mode with a scan speed of 0.02° (2 *θ*)/min. Phases were identified by matching experimental patterns to the JCPDS powder diffraction file [31]. Lattice parameters were evaluated from all reflections appearing in the range of 2*θ* = 10–70°, by means of the UNITCELL program [32]. Surface morphology of the samples was studied by scanning electron microscopy (SEM) using a Jeol JSM-840 instrument operated at 3 kV. The specimens observed were sputter-coated with a 4–8 nm layer of Au/Pt. The amount of CuO/Cu_2_(OH)_3_NO_3_:(Co^2+^/Fe^3+^) in the composite was determined from inductive coupled plasma emission spectrometry data (Vista AZ CCD, registered simultaneous ICP-AES).

### 3.4. Catalytic CO Conversion

Prior to the testing of catalytic activity in the CO conversion, specific surface area (*S*_sp_) of the samples was determined by Ar desorption at 77 K, using a Brunauer-Emmett-Teller area analyzer.

The catalytic activity was examined in a stream U-type microreactor at normal atmospheric pressure. The sample powder (*m* = 0.5 g) was placed on a calcined glass-fibers plate, fixed in the middle of the microreactor. The sample powder forms a uniform catalyst bed of 8 mm height. The composition of the gases mixture was monitored in the microreactor outlet after switching in the inlet from He to a mixture 20 vol % O_2_ + 2 vol % CO + 78 vol % He. The gases were provided by Iceblick Speciality Gas Co. (Odessa, Ukraine, >99.99%) and used as received. The gas flow of 1.67 × 10^–6^ m^3^·s^–1^ used through the study was controlled by mass-flow controllers. The reaction temperature was monitored by a Chromel thermocouple placed in the catalyst bed. During the studies, the reaction temperature was varied from 293 K to whatever temperature was necessary to achieve complete CO conversion to CO_2_ and then reduced to a temperature yielding low conversion, typically 300 K.

This procedure was then repeated for at least two cycles during each run to ensure that the catalyst had reached a steady temperature at a certain conversion of CO to CO_2_. During the initial heating period, care was taken to allow the temperature to equilibrate before a sample of the outlet gas was taken. The composition of the effluent gas mixture was analyzed on-line by a LHM-8MD gas chromatograph (Russ. Gaschrom Co.), the gases (O_2_, CO, CO_2_) were separated in a C/NiSO_4_ capillary column and detected with a thermal conductivity detector. CO and O_2_ conversions were calculated from the differences between their inlet and outlet concentrations, the *t*^100%^ value was used as a measure of the catalytic activity. The pure TEG was used as a reference material, which temperature at 90% conversion of CO to CO_2_ was used to be compared with that of composites.

### 3.5. Characterization of Adsorbed Species over Composites Taken after CO Conversion

The surface state of the composites was examined by temperature programmed desorption mass-spectrometry (TPD MS). TPD MS measurements were performed by heating of the samples taken after catalytic test in a high vacuum (*p* < 10^–4^–10^–5^ Pa) in quartz cell using a linear heating regime with 0.17 K·s^–1^ rate to detect adsorbed species. Evolving products were analyzed using a МХ 7304 А mass-spectrometer as a detector of desorbed species. The TPD MS profiles of Н_2_O, CO_2_, O_2_ and atomic oxygen were collected and the activation energy of CO_2_ desorption (*E*_d_) was determined from the profiles by the Amenomija-Cvetanovic method [33].

## 4. Conclusions

The combined use of several characterization techniques allows us to estimate the factors affecting the catalytic activity of the composites and leads to the following conclusions:

1. The TEG impregnation with nitric or sulphuric acids with subsequent thermal shock allows achievement of complete graphite exfoliation necessary for improvement of the composites’ activity.
2. The activity depends on the CuO/Cu_2_(OH)_3_NO_3_:(Co^2+^/Fe^3+^) component content. The high activity of (TEG)/CuO/Cu_2_(OH)_3_NO_3_:(Co^2+^/Fe^3+^) composites is attributed to higher dispersion over the TEG, the latter being achieved by means of the wet impregnation under ultrasonic agitation.
3. Comparative studies on CO oxidation reveal that Cu_2_(OH)_3_NO_3_ phase, registered in the content of the composites catalysts, markedly improves their activity.
4. The decrease in CO oxidation activity has been ascribed to the decomposition of Cu_2_(OH)_3_NO_3_, which was more active than CuO phase, and prevailing over CuO in the composite content. Catalyst with 57% of CuO/Cu_2_(OH)_3_NO_3_:(Co^2+^/Fe^3+^) showed the best conversion efficiency of 100% at a temperature as low as 403 K for the CO conversion to CO_2_. Relation between *α*_2_ and *α*_3_ states of CO_2_, desorbing from the surface of the composites, correlates with the catalytic activity. The high content of α_2_ state registered in the TPD MS spectra is a feature of the most active catalysts.
